# The contribution of bacterial genome engineering to sustainable development

**DOI:** 10.1111/1751-7915.12784

**Published:** 2017-08-03

**Authors:** Daniel R. Reuß, Fabian M. Commichau, Jörg Stülke

**Affiliations:** ^1^ Department of General Microbiology Institute of Microbiology and Genetics Georg‐August University Göttingen Grisebachstr. 8 D‐37077 Göttingen Germany

## Abstract

The United Nations’ Sustainable Development Goals define important challenges for the prosperous development of mankind. To reach several of these goals, among them the production of value‐added compounds, improved economic and ecologic balance of production processes, prevention of climate change and protection of ecosystems, the use of engineered bacteria can make valuable contributions. We discuss the strategies for genome engineering and how they can be applied to meet the United Nations’ goals for sustainable development.

Several of the United Nations’ Sustainable Development Goals (SDG) can be addressed by biotechnological approaches. Today, it is possible to produce value‐added compounds for the health care, food additive, animal feedstock, petrol and chemical industry sectors by or with the help of genome‐engineered microorganisms (SDGs No. 2 and 3). For this purpose, novel heterologous pathways can be implemented and combined with existing metabolic functions. In this way, tailor‐made organism for industrial applications can be build up. Genome engineering has been used for the efficient production of ethanol, vitamins, enzymes, carbohydrates and chemical precursors in the bacteria *Bacillus subtilis* and *Escherichia coli* (Romero *et al*., 2007; Liu *et al*., 2013; Qi *et al*., 2015; de Oliveira and Nicholson, 2016). Once fully established, biotechnological production processes can have improved economic and ecologic balances as compared to classical chemical synthesis or extraction techniques (SDG No. 12). For instance, the production of riboflavin (vitamin B_2_) with *Ashbya gossypii* reduced the production and environmental protection costs and the CO_2_ emission by 43% and 30%, respectively, as compared to the chemical synthesis (Wenda *et al*., 2011; Revuelta *et al*., 2016). Moreover, modified microorganisms can be used to fight major ecological challenges such as the climate change and can contribute to the protection of ecosystems (SDGs No. 13 and 15). Recently, a highly efficient synthetic CO_2_‐fixation pathway was designed *in vitro*. The implementation of this pathway into bacteria can contribute to the SDGs in two distinct ways. First, this pathway consumes the greenhouse gas carbon dioxide (and therefore reduces its impact on climate change), and second, CO_2_ is converted into organic compounds to build up biomass. In combination with additional genome engineering, e.g. photosynthetic processes and further pathways for carbohydrate metabolism, self‐sustainable microbial production platforms can be envisioned today (Erb and Zarzycki, 2016; Schwander *et al*., 2016). Furthermore, modified microorganisms are of high interest for wastewater treatment. Industrial wastewater can be contaminated with an inexhaustible variety of harmful compounds, such as radioactive particles, heavy metals, solvents, antibiotics and other organic compounds. Efficient biotechnological applications require highly resistant microorganisms with natural or engineered degradation pathways. For example, acetonitrile, a water‐soluble substrate that can be converted to toxic hydrogen cyanide in human and animal cells, is a common contaminant in wastewater of the chemical industry (Greenberg, 1999; Robles, 2014). It has been shown that the biofilm‐forming strain *B. subtilis* N4‐pHT01‐*nit* strain expressing the *Rhodococcus rhodochrous* nitrilase Nit was not only resistant to acetonitrile, but it was able to degrade the molecule (most of the added 800 mg l^−1^ within 24 h; Li *et al*., 2016b). This approach shows how simple a biotechnological application can be. However, the replacement of an established chemical synthesis takes years of research and might never reach the same economic efficiency.

Taken together, the possible applications of genetically modified organisms and their contribution to the SDGs are numerous and the rising awareness for the protection of our ecosystems will drive the further research for successful biotechnological applications in the future.

## Construction of tailor‐made bacteria by genome engineering

Genetically modified organisms can play a major role in meeting our high demand for refined industrial products and to fight major threats to ecosystems. Nevertheless, the industrial application of genome‐engineered strains requires (i) labour‐intense analyses of literature, (ii) screening of databases, (iii) *in vitro* an *in vivo* construction of the desired pathways with the right choice of organisms, (iv) protein engineering for altered enzyme specificity, and (v) small laboratory‐size application and finally scale‐up processes for industrial implementations. A major bottleneck in this workflow is the successful construction of the desired strains. Five different attempts with increasing complexity of genome engineering are conceivable: (i) targeted evolution of organisms, (ii) modification of native pathways, (iii) introduction of natural non‐native pathways, (iv) introduction of synthetic pathways, and (v) introduction of natural and synthetic pathways into synthetic organisms. Targeted evolution experiments and simple genetic modifications can easily be performed with a wide range of microorganisms and have demonstrated their power in several studies (Conrad *et al*., 2011; Portnoy *et al*., 2011; Dragosits and Mattanovich, 2013). However, the design, construction and implementation of synthetic pathways or even entire synthetic organisms require a deep understanding of all levels of cellular functions and a variety of different molecular techniques.

**Figure 1:**
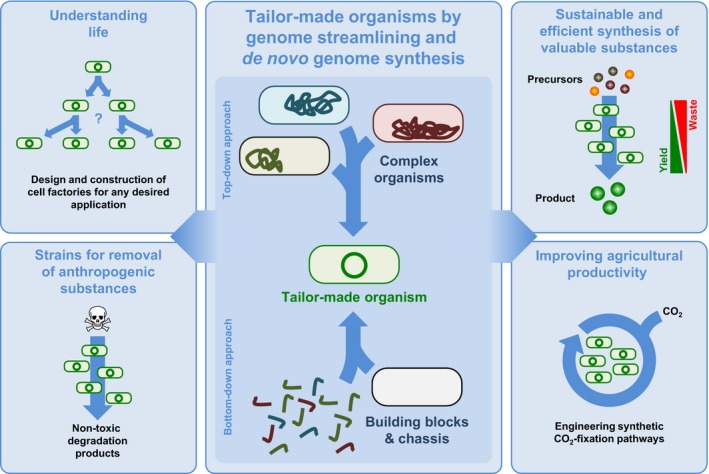
Approaches for the application of genome engineering to construct tailor‐made organisms that help to achieve sustainable development.

In the past decades, substantial technical progress has been made and it is possible to analyse biological samples globally (genome, transcriptome, proteome, metabolome and interactome), to synthesize DNA oligonucleotides chemically at low costs and to build up synthetic operons *in vivo* and *in vitro* efficiently. We can ligate multiple large DNA fragments at once by Gibson assembly, the ligase cycling reaction (LCR) or by the sequence and ligation‐independent cloning method (SLIC) *in vitro* (Li and Elledge, 2007; Gibson *et al*., 2009; de Kok *et al*., 2014). Furthermore, the access to circular yeast artificial chromosomes together with the efficient recombination machinery of *Saccharomyces cerevisiae* has been used to assemble large synthetic DNA fragments and even complete genomes *in vivo* (Gibson *et al*., 2008, 2009; Karas *et al*., 2012; Noskov *et al*., 2012). These techniques open the door for the construction of entire synthetic organisms in bottom‐up approaches as chassis for industrial production platforms (see Fig. 1). Very recently, the first organism with an *in silico* designed and completely chemically synthetized chromosome has been constructed in this way (Gibson *et al*., 2010; Hutchison *et al*., 2016). Alternatively, microorganisms can be streamlined by step‐wise genome reduction and genome engineering as well. The chromosomes of existing microorganisms are reduced by the introduction of dozens of marker‐free deletions, which is called the top‐down approach (Fig. 1). Today, a variety of marker‐free deletion systems is available and most of them rely on the selection/counter‐selection principle. For this purpose, a deletion construct is introduced into the chromosome, which can be selected for auxotrophic or resistance markers. The subsequent removal of this construct can be achieved by selection against toxic counter‐selection marker, e.g. *mazF, upp* or *manP* (Fabret *et al*., 2002; Morimoto *et al*., 2009; Wenzel and Altenbuchner, 2015). Moreover, marker‐free deletions can be performed using the Cre/loxP and the Flp‐FRT systems. Both systems rely on the introduction of palindromic sequences adjacent to the deletion region. The recombinases Cre and Flp can bind these sequences and recombine the DNA strands resulting in a deletion between the palindromic sequences (Sternberg and Hamilton, 1981; Zhu and Sadowski, 1995). Such deletion systems have been used to reduce the chromosomes of a variety of prokaryotes, e.g. *E. coli, B. subtilis, Pseudomonas putida, Streptomyces avermitilis* and *Corynebacterium glutamicum* (Morimoto *et al*., 2008; Komatsu *et al*., 2010; Leprice *et al*., 2012; Hirokawa *et al*., 2013; Unthan *et al*., 2015; Reuß *et al*., 2017). However, the most promising technique for genome engineering is the CRISPR‐Cas system. This recently discovered mechanism allows deletion, modification and insertion of genomic regions in all three domains of life (Jinek *et al*., 2012; DiCarlo *et al*., 2013; Jiang *et al*., 2013; Li *et al*., 2016a). In principle, a RNA composed of a site‐specific and a Cas9‐recruiting part guide the endoribonuclease Cas9 to a specific locus. The enzyme introduces double‐strand breaks, which can be repaired by non‐homologous end‐joining or by homologous recombination, which in turn leads to a deletion or an insertion, if an additional DNA fragment with homologous flanks is provided (Zheng *et al*., 2014).

Many genome‐reduced bacteria show beneficial traits, such as higher heterologous enzyme or higher amino acid production, which makes them especially interesting for industrial applications (Mizoguchi *et al*., 2008; Trinh *et al*., 2008; Lee *et al*., 2009; Manabe *et al*., 2013). Nevertheless, genome engineering often leads to undesired effects. In particular, altered gene expression and biosynthesis of enzymes can cause accumulation of toxic intermediates, interference with other pathways and changes of the redox state of the cell (Kokura *et al*., 1999; Hong and Lee, 2002; Arévalo‐Rodríguez *et al*., 2004; McGary *et al*., 2013; Commichau *et al*., 2015). These side‐effects are major challenges in genome engineering and have to be addressed individually. The expression levels of operons can be modified with the right choice of promoters and ribosomal binding sites (Guiziou *et al*., 2016; Rosenberg *et al*., 2017), and the interference with other pathways can be circumvented by further genome reduction or evolution experiments (Manabe *et al*., 2011, 2013; Commichau *et al*., 2015). However, we still do not fully understand even intensively studied organisms like *B. subtilis* and *E. coli*, which in turn precludes the prediction of possible undesirable phenotypes in many cases.

In principle, we have access to all required molecular techniques and tools for genome engineering to build any synthetic pathway and even entire synthetic organisms. Furthermore, we can analyse the specific and the global impact on the cellular functions when modified or heterologous pathways are implemented. Thus, the application of genome engineering techniques can be expected to make a major contribution to achieve the United Nation's Sustainable Development Goals.

## Conflict of interest

None declared.
